# The Thermoviscosifying Behavior of Water-Soluble Polymer Based on Graft Polymerization of Pluronic F127 with Acrylamide and 2-Acrylamido-2-methylpropane Sulfonic Acid Sodium Salt

**DOI:** 10.3390/polym11101702

**Published:** 2019-10-16

**Authors:** Hongping Quan, Luyao Xie, Xin Su, Yujun Feng

**Affiliations:** 1 Oil & Gas Field Applied Chemistry Key Laboratory of Sichuan Province, School of Chemistry and Chemical Engineering, Southwest Petroleum University, Xindu 610500 China; 2 Polymer Research Institute, State Key Laboratory of Polymer Materials Engineering, Sichuan University, Chengdu 610065, China

**Keywords:** thermoviscosifying, water-soluble polymer, polyether

## Abstract

A new concept of thermoviscosifying polymers is proposed to address the problems about decreasing viscosity of polymer solution under high temperatures. However, existing thermoviscosifying polymers have complicated synthesis processes and high costs, and both of them restrict the wide practical applications of thermoviscosifying polymers. Although polyethers have the characteristics of thermal gelatinization, they just display thermoviscosifying behaviors only under extremely high concentrations (>15 wt %). Therefore, the graft copolymerization of the commercialized Pluronic F127 (PEO_100_-PPO_65_-PEO_100_) with acrylamide and 2-acrylamide-methylpropionic acid sodium salt was studied here. A series of graft modified polyether polymers were prepared and it was expected to get thermoviscosifying polymers with high molecular weights and low association temperatures. Several factors on thermoviscosifying behaviors were investigated, such as polymerization condition, polymer concentration, hydrophilic monomer, molecular structure and molecular weight. It was also proven that the apparent viscosity of polymer solution is influenced by polymer concentration, molecular weight of polymer, and content of anion groups.

## 1. Introduction

Petroleum has been an important source for sustainable economic development. The chemical flooding for enhanced oil recovery (EOR) depending on water-soluble polymer is one of main methods to increase the output of crude oil [1]. It mainly expands the sweep efficiency by increasing viscosity of the flooding fluid and thereby improves the recovery efficiency [2]. Partially hydrolyzed polyacrylamide (HPAM) and its derivatives are the mostly used water-soluble polymers in EOR. Although this type of polymers has merits like easy accessibility of raw materials, low cost and good water solubility, they also have some disadvantages: (1) decreasing viscosity of their aqueous solutions with rising temperature; (2) when the temperature is higher than 75 °C, acylamino groups in molecular structures may be further decomposed into carboxylic acids which may make complexation and thereby cause phase separation by high-valence metal ions (e.g., Ca^2+^ and Mg^2+^). The viscosity is hard to be enhanced significantly under practical conditions, which makes it difficult to meet the application requirements [3,4,5].

Considering poor performances of HPAM, some researchers have studied a series of new water-soluble polymers for EOR. In particular, Hourdet team [6,7] proposed the novel concept of “thermoviscosifying polymers” (TVPs) for the first time. Thermoviscosifying water-soluble polymer refers to introducing side chains with low critical solution temperature (LCST) into the backbones of water-soluble polymer, as the introduced side chains can response to temperature stimuli. Therefore, the aqueous solutions of the grafted polymers have the unique thermal association features when exceeding the critical association concentration (CAC). When temperatures (T) < LCST, the whole polymers are manifested as hydrophilicity because of the strong hydrogen-bond interaction between side chain of polymer and water molecules; when T > LCST, the whole polymers display as amphipathic, and the reason is that hydrophobic association occurs between side chains of polymer, which is manifested by hydrophobicity at that time [8]. Due to the hydrophobic association effect between side chains, a physical crosslinking network structure is formed between the main chains, increasing hydrodynamic volume. Macroscopically, the viscosity of the aqueous solution increases when temperature rises, which is known as thermoviscosifying behaviors. Meanwhile, above LCST, the viscosity of the TVP saline solution increases as temperature increases, while the viscosity of HPAM solution decreases with increasing salinity [9,10]. Due to these special properties, namely the high temperature and salinity do not diminish the viscosity of their aqueous solutions, TVPs have attracted much attention from oil and gas industries.

TVP is a type of critical intelligent water-soluble polymers possessing important potential application values as an oil-displacing agent. Currently, the most TVPs generally contain functional groups in their molecular structures [11,12,13]. They can mainly be divided into three types: (1) poly(N-isopropylacrylamide) (PNIPAM) and its derivatives [14]; (2) cellulose derivatives [15]; (3) polyether derivatives [16]. However, the present TVPs still have some weaknesses. For example, PNIPAM achieves apparent thermoviscosifying effect, but it gains low temperature sensitivity and high cost, which restricts its industrial usage. Although cellulose derivatives are nontoxic and biocompatible, they are easy to make phase separation under high temperature. As a commercial polyether, Pluronic is a type of triblock copolymers with poly(ethylene oxide) and poly(propylene oxide) (PEO-PPO-PEO), which is cheap and their LCST is changeable, but it only shows thermoviscosifying performance under high polymer concentration (>15 wt %) [17]. These inadequacies blocked practical usage of TVPs and inspire us scouting novel methods to obtain TVPs.

Introducing side chains of polyether into regular water-soluble polymers is a new strategy to increase thermoviscosifying behavior without increasing the polymer concentration [9]. Compared with single chains of PEO, PPO or PEO-PPO diblock copolymer, the triblock copolymers of PEO-PPO-PEO possesses wide and controllable LCST. Different structures of PEO-PPO-PEO triblock copolymers have different rheological properties. It is found that Pluronic F127 (PEO_100_-PPO_65_-PEO_100_) with a linear molecular structure shows the best rheological behavior. There are several labs contributing the research about thermoviscosifying using Pluronic F127. For obtaining a thermothickening effect at low polymer concentrations, the Bromberg team prepared a series of TVPs by grafting different Pluoronic polyethers onto PAM or PNaAA by dispersion polymerization [18,19,20]. They obtained F127-g-PAA with MW as high as 3.61 × 10^6^ g⋅mol^−1^, and thus only at 0.5 wt %, a typical thermoviscoelastic response was observed [18]. Tam et al. found that poly(lactic acid) (PLA) could be grafted to both ends of Pluronic F127 (PEO-PPO-PEO) to produce novel amphiphilic PLA-F127-PLA block copolymers [21]. Lam et al. aimed to study the association and interaction behavior of Pluronic polymers grafted with poly(vinylpyrrolidone) and noticed that the thermal responsiveness of the copolymer was not affected by the graftin [22,23]. Alexandridis et al. discovered the phase behavior of Pluronic F127 with water and organic solvents, revealing the block copolymer structural polymorphism is modulated by the solvent preference to locate in different domains of the block copolymer microstructure [24]. Our group also reported thermoviscosifying grafted triblock copolymers with F127 using inverse emulsion polymerization [25]. Nevertheless, powder forms have inherent advantages: powders are more convenient for long-distance logistic transportation. Moreover, for discovering the mechanism of thermoviscosifying with F127 further, we prepared a kind of graft polymer using a neutral monomer and an anionic monomer. In this study, a series of new anionic TVPs containing sulfonates were prepared based on Pluronic F127 through graft polymerization; some water-soluble monomers, such as acrylamide (AM) and 2-acrylamido-2-methylpropane sulfonic acid sodium salt (NaAMPS), were used to improve the solubility and hydrophilicity of the prepared polymers. It is expected to achieve good thermoviscosifying performance under low polymer concentration.

## 2. Materials and Methods

### 2.1. Materials

2-Acrylamido-2-methylpropane sulfonic acid (AMPS), acrylamide (AM), dodecane, hexane, NaOH and NaCl were all analytical grade and purchased from Chengdu Kelong Chemical Reagent Co. Ltd, Chengdu, China. NaAMPS was prepared by neutralizing AMPS with equimolar of NaOH. The commercial Pluronic F127 was purchased from Sigma-Aldrich and used without further treatment. 2,2′-Azobis(2,4-dimethylvaleronitrile) (V52) was bought from J&K Chemicals Ltd., Beijing, China. Lauroyl peroxide (LPO) was purchased from Sigma-Aldrich Ltd. N_2_ (99.998%) was supplied from the Tianyuan Gas Company, Chengdu, China. The deionized water used in this study (conductivity, κ = 18.25 μS·cm^–1^) was triple distilled using a quartz water purification system.

### 2.2. Synthesis of Polymers

**Scheme 1:**
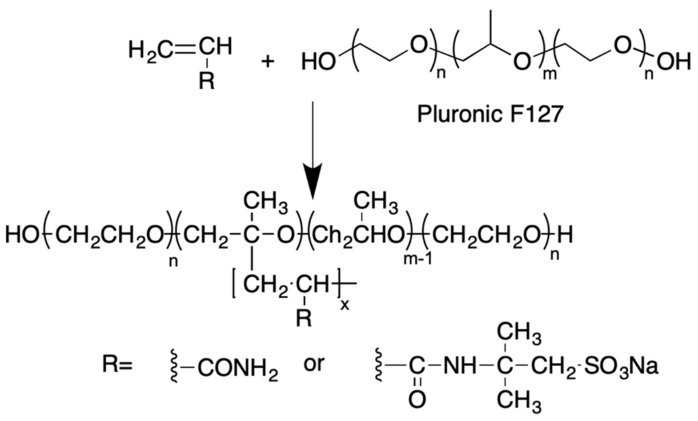
Synthesis route for graft copolymerizations of polyethers.

The synthesis route of graft polymers is shown in Scheme 1. Firstly, water-soluble monomer (AM or/and NaAMPS), Pluronic F127 and water (100 ml) under the designed amount were added into a 500 mL flask with four necks. The mixture was stirred continuously at a constant speed until dissolving was completed. Next, dodecane (100 mL) with containing LPO (0.03 g, 0.075 mmol) and V52 (0.01g, 0.04 mmol) was added and the mixture in the flask was stirred for further 0.5 h. N_2_ was supplied for removing O_2_ throughout the whole polymerization process. The mixture was heated to 70 °Cat a rate of about 1~2 °C /min. This temperature (70 °C) was kept for 4 h and then cooled to room temperature. The prepared white solid in the flask were filtered and collected, and they were cleaned by hexane. Finally, the white powder was dried to a constant weight in vacuum under 40 °C. The aqueous solutions of the obtained polymer were charged in a beaker with a magnetic stirrer and dialysis tubing (MWCO 2000, Aldrich, Beijing, China). Deionized water (50 mL) was fed to the beaker and the mixture was stirred for 6 d; the water in the beaker was renewed every day. The purified solution was treated by freezing-drying at −20 °C for 48h and the yield of the polymer was measured by gravimetry. Detailed information for the series of prepared polymers using different monomers is shown in Table 1 and Table 2.

### 2.3. Characteristics

FT-IR: 380 Fourier transform infrared spectrometer (Nicolet Instrument Technologies, Inc, Madison, WI, USA) was applied to analyze polymer samples and the scanning range was 4000~400 cm^−1^. FT-IR was used once per polymer sample.

NMR: purified polymers were dissolved in D_2_O and then tested by the Ascend TM 800 MHz NMR instrument (Bruker Corporation, Billerica, MA, USA). ^1^H NMR and ^13^C NMR were scanned under 800 MHz for 4 s and 6.5 h under 25 °C.

Intrinsic viscosity testing: Viscosity of polymer aqueous solutions is generally tested by an Ubbelohde capillary viscometer (CV003-006, Thomas Scientific, Swedesboro, NJ, USA). Polymer solution is diluted and the delivery time of five solutions with different concentrations was measured. Based on deduction to “0” concentration by combining the Huggins equation, Equation (1) [18], and the Kraemer equation, Equation (2) [19], the intrinsic viscosity [*η*] could be calculated:

(1) $$\frac{\eta_{\mathrm{sp}}}{C}=[\eta ]+K_{H}{\left[\eta \right]}^{2}C$$

(2) $$\frac{\ln\eta_{r}}{C}=[\eta ]+K_{K}{\left[\eta \right]}^{2}C$$

where *K*_H_ and *K*_K_ are Huggins constant and Kramer constant, respectively.

Static light scattering (SLS): the formamide and water were mixed at a mass ratio of 1:1 and the mixture was used as the solvent. The polymer samples were dissolved with different concentrations ranging between 1000~2000 mg/L, followed by 15 min of centrifugation at a rate of 10,000 rpm. The solutions were filtered by 0.22 μm needle filters and 0.80 μm needle filters successively. Samples were tested by the BI-200SM scattering light instrument (Brookhaven Instruments Corporation, Holtsville, NY, USA) under the following conditions: the excitation wavelength was 532 nm; methylbenzene was used as the reference liquid; the testing angle ranged between 25°–140°. The weight-average molecular weight (M_w_) of the polymer was calculated by Zimm/Berry plot [20]. The refractive index increment (dn/dc) value of the solutions is 0.170 ± 0.002 mL g^−1^, which was measured by a differential refractometer (1260 Infinity, Agilent, Santa Clara, CA, USA).

Dynamic light scattering (DLS): the solution was tested using Malvern Zetasizer Nano ZS. In the test, impurities in the testing samples were removed with a 0.8 μm probe filter. Each sample was tested at least three times.

Rheological property: rheological properties were tested by a rotating viscometer (Physica MCR 301 rheometer, Anton Paar, Graz, Austria) and its equipped CC27 rotor/shaft system. The temperature controlling accuracy in the rheometer could reach 0.01 °C. All samples were stabilized under a constant temperature (20 °C) for 15 min before the test.

Gel permeation chromatography (GPC): GPC measurements (Shodex 3000 GPC system consisting of an HPLC pump) were done to measure molecular weight of samples. This GPC was equipped with a Waters separation module, including four polystyrene columns with pore sizes of 100, 500, 103 and 104 Å, and a differential refraction detector at 40 °C. Tetrahydrofuran was used as the eluent with a flowrate of 0.3 mL/min. Molecular weights were determined using polystyrene standards over the range of 870−875,000 g·mol^−1^.

## 3. Results and Discussion

### 3.1. Molecular Structure of Prepared Polymers

Pluronic F127 was grafted with AM or/and NaAMPS respectively, to obtain a series of binary copolymerized TVPs based on polyether. The molecular structures of the prepared TVPs were characterized by FT-IR and NMR, to determine the proportion of grated side chains in the TVPs; the intrinsic viscosity of the TVPs aqueous solutions is established; the M_w_ of the TVPs is tested by light scattering.

#### 3.1.1. FT-IR

**Figure 1:**
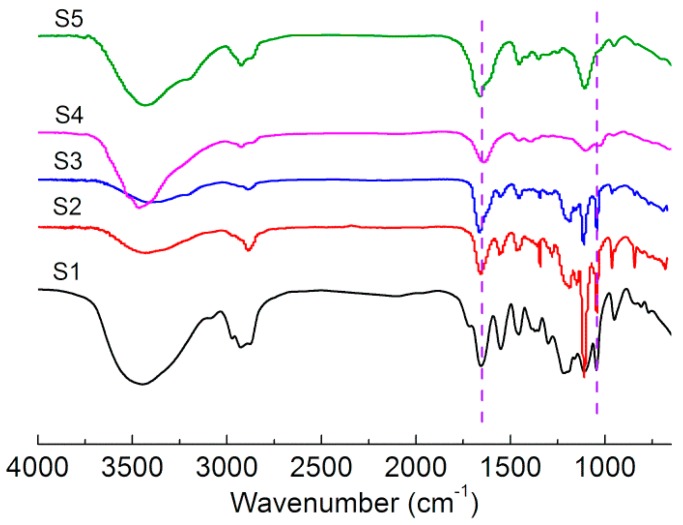
FT-IR spectra of the five prepared polymers.

All the grafted copolymers of Pluronic F127 were characterized by FT-IR. The FT-IR spectra of polymers S1, S2, S3, S4 and S5 (Table 1) are shown in Figure 1. Based on comparison of FT-IR spectra of five polymers, the absorption peak of hydroxyls in Pluronic F127 segment locates at 3400~2900 cm^−1^, while the absorption peak of ether bond is at 1110~1250 cm^−1^. Moreover, the asymmetric stretching vibration absorption peak of S=O is about 1050 cm^−1^. A stretching vibration absorption peak of C=O is produced at 1650 cm^−1^. This demonstrates preliminarily that Pluronic F127 is grafted successfully with AM or/and NaAMPS to prepare S1, S2, S3, S4 and S5.

#### 3.1.2. ^1^H NMR Spectra

**Figure 2:**
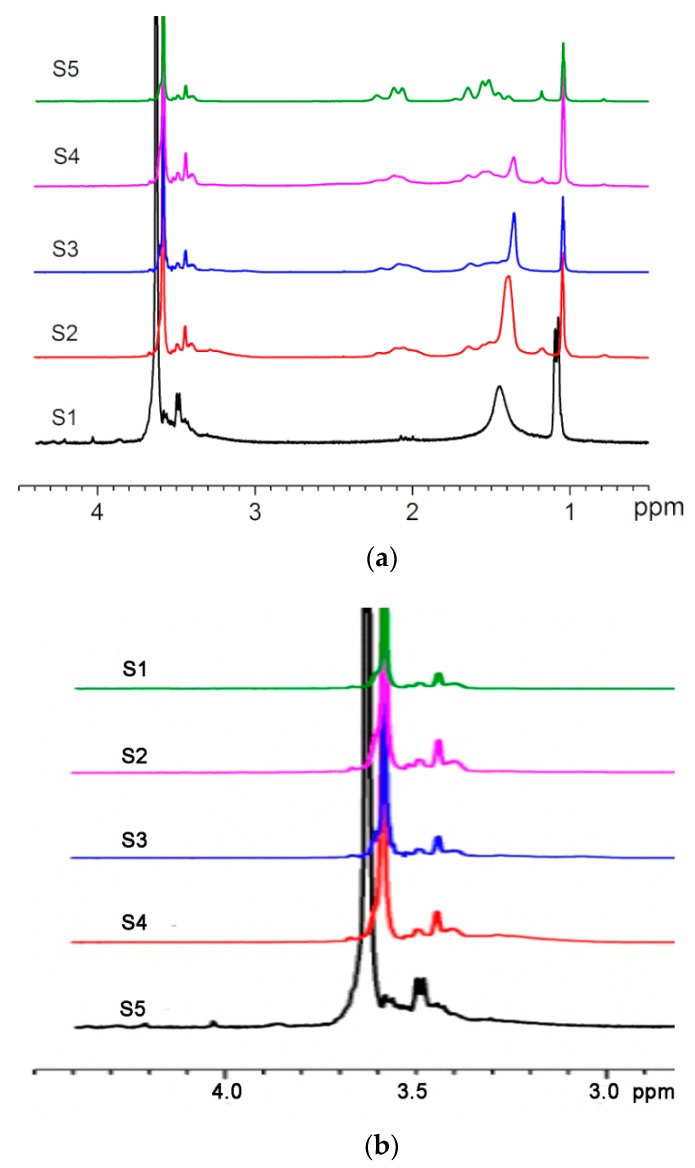
^1^H NMR spectra for the five prepared polymers: (**a**) 1.0–4.0 ppm; (**b**) 3.0–4.0 ppm.

^1^H NMR spectra of S1, S2, S3, S4 and S5 are displayed in Figure 2. The chemical shift of the groups conforms to the theoretical value. The chemical shift of 1.0~1.1 ppm is the absorption peak of methyl in polyether. The chemical shift of 3.5~3.7 ppm is the absorption peak of polyether. The chemical shift of 1.4~1.8 ppm is the characteristic peak of –CH_2_– and –CH_3_ in NaAMPS. The chemical shift of 2.0~2.2 ppm is the absorption peak of –CHCO– in NaAMPS. The chemical shift of 3.0~3.4 ppm is the absorption peak of –CH_2_– in AMPS (Figure 2b). The chemical shift of 1.5~1.8 ppm is the absorption peak of AM and NaAMPS. These chemical shifts can prove the graft reaction of F127, AMPS and acrylamide.

**Figure 3:**
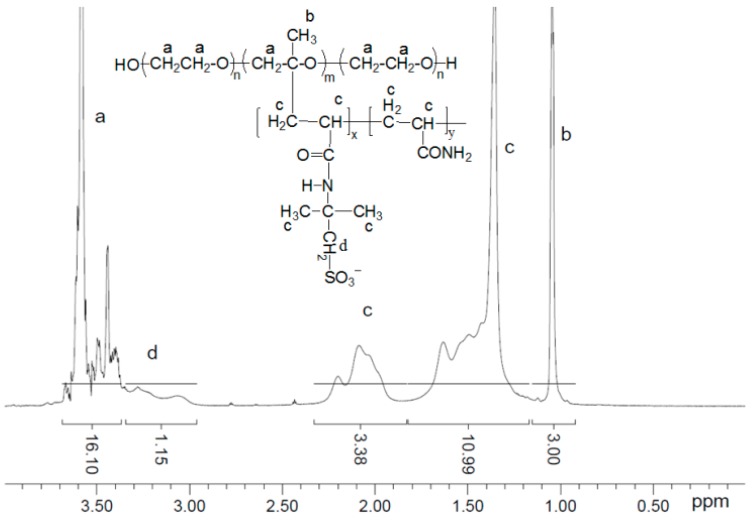
^1^H NMR spectrum of the polymer S3.

According to Scheme 1, the number of H atoms in methyl groups from Pluronic F127 remained the same before and after the grafted polymerizations. Moreover, one side chain was produced by replacing one H atom on the polyether chain. Therefore, the H atom absorption peak area (*S*_a_) of CH and CH_2_ in grafted F127 is decreased. The graft ratio can be defined as the degree of reduction:

(3) $$B=\frac{S_{a}-{S_{a}}^{\prime }}{S_{a}}\times 100\%$$

where B is graft ratio; *S*_a_ and *S*_a_′ are the integral area sums of CH and CH_2_ in Pluronic F127 before and after the grafted polymerizations. The polymer S3 is taken as the example for showing the procedure of calculating using Equation (3) and it has been the same for all others samples. The ^1^H NMR spectrum of the polymer S3 (Figure 3) shows *S*_a_′ = 16.10, while *S*_a_ is 16.95 before the grafted polymerization. Based on the Equation (3), the graft ratio is calculated as B = 5.01%.

Similarly, the proportion of NaAMPS in grafted polymers also can be calculated by integral area in ^1^H NMR spectra. The absorption peaks on ^1^H NMR spectra of NaAMPS and AM overlap and cannot be distinguished well. But, according to *S*_b_, *S*_c_ and *S*_d_ (the peak areas of b, c and d in Figure 3), the chemical formula of polymers could be calculated by using F127 (1 mol) as the reference. The polymerization degree of NaAMPS in polymer S3 the is 37, namely n_(AMPS)_ = 37, which was calculated by the Equation (4):

(4) $$\frac{S_{b}}{S_{d}}=\frac{3\times 65}{2\times n_{\left(\mathrm{AMPS}\right)}}$$

where *S*_b_ and *S*_d_ are the integral area sums of peaks b and d shown in Figure 3.

#### 3.1.3. ^13^C NMR Spectra

**Figure 4:**
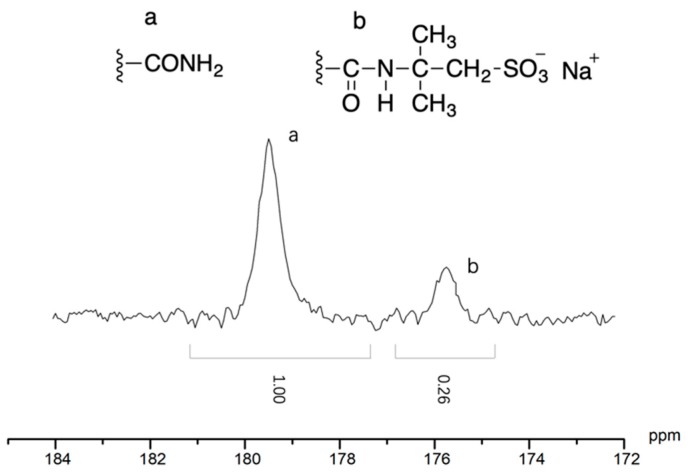
^13^C NMR spectrum of the polymer S3.

From Figure 4, it can be seen that the chemical shift of C atom in carbonyl conforms to the theoretical value. For the polymer S3, the chemical shift 179.0 ppm is the absorption peak of C atom of carbonyl in AM, while the chemical shift of 176.0 ppm represents the carbonyl groups in NaAMPS. Using Equation (4) and Equation (5), it was deduced that the polymerization degree in polymer S3, n_(AM)_ is 142. Therefore, the chemical formula of S3 can be expressed by F127-AMPS_37_-AM_142_.

(5) $$n_{\left(\mathrm{AM}\right)}=\frac{S_{\left({\mathrm{CONH}}_{2}\right)}}{S_{\left(\mathrm{CONH}\right)}}\times n_{\left(\mathrm{AMPS}\right)}$$

where *S*_(CONH2)_ and *S*_(CONH)_ are the integral area of –CONH_2_ (from AM) and –CONH– (from NaAMPS) shown in ^13^C NMR spectra.

In the same way, the chemical formula of the five prepared polymers were obtained and shown in Table 1.

#### 3.1.4. SLS Results

Table 3 shows the molecular weight of the prepared polymers. Most of them can be tested by GPC (Figure S1), but the molecular weight of S4 is so large that SLS has to be used. The association effect has to be eliminated before measuring the M_w_ of hydrophobic association polymers by SLS; otherwise, the tested M_w_ is hardly their real molecular weight. The reason is that the hydrophobic association polymers do not scatter as single molecules in aqueous solutions, but exist as aggregates. Although NaCl solution can eliminate polyelectrolyte effect, it cannot make the association effect disappear. However, formamide can eradicate association effect effectively. It is found the mixture of formamide and water (mass fraction 1:1) is the good solvent for the prepared grafted polymers.

**Figure 5:**
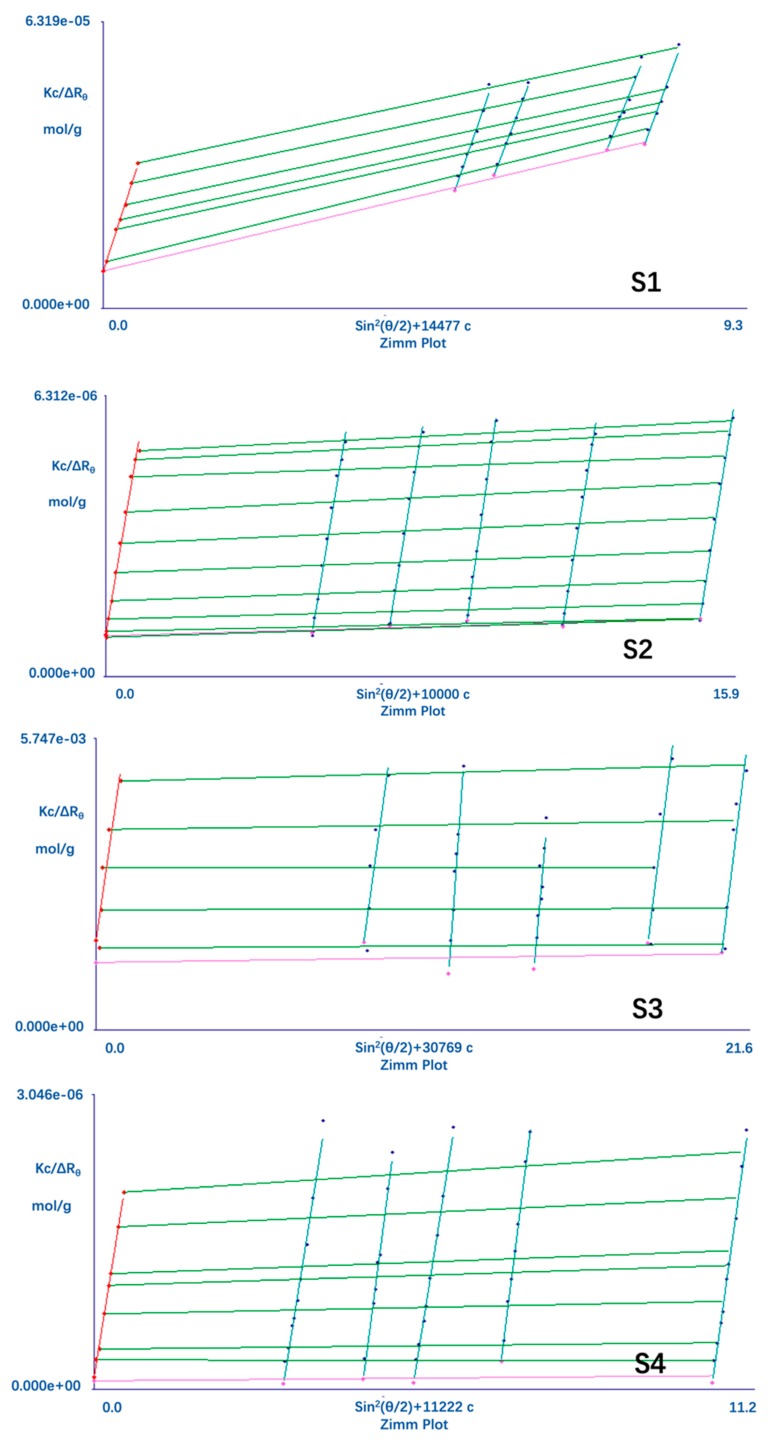

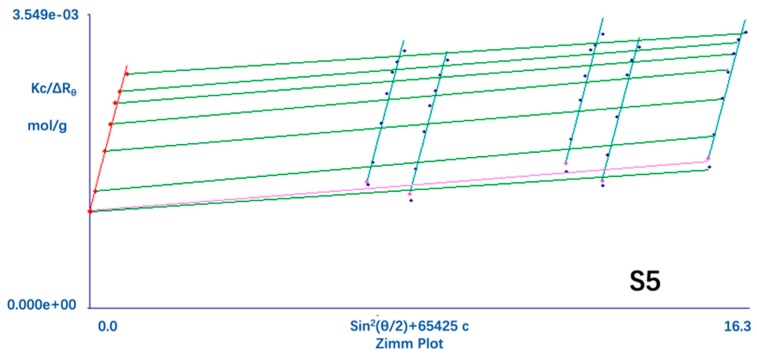
Zimm plots for S1, S2, S3, S4 and S5 in mixed formamide/water (1:1).

It is reported that the concentration extrapolation line in the Zimm plot bent upward when testing polymers with high M_w_. The second Virial coefficient (A_2_) through concentration extrapolation according to the trend of curves is shown in Table 3. To solve the problem, the Berry equation was applied: a series of parallel straight lines was obtained by extrapolation of concentration and angle [20]. Slopes of these parallel straight lines were A_2_ and the intercepts were M_w_. In Figure 5, all the slopes are positive, and it implies that all the values of A_2_ are positive. This indicates that the mixture of formamide and water is a good solvent. The weight-average molecular weights of S1, S2, S3, S4 and S5 are shown in Table 1. It is notable that M_w_ of S4 is pretty high (~15 million). The radius of gyration (R_g_) was tested and Table 3 demonstrates that S4 has the largest R_g_, which matches the result of hydrodynamic radius measured by DLS (Figure S2).

Additionally, the Huggins constant K_H_, calculated from the Huggins equation (Equation (1), Figure S3), is also an indicator for evaluating the solvent thermodynamic quality based on its intimate correlation with A_2_. As noted in Table 3, the calculated K_H_ values for the three polymers used here are in the range of 0.2–0.4, suggesting that the solvent (mixed formamide/water, 1:1) used is favorable.

### 3.2. Viscosity Properties of Polymer Solution

The rheological method is used to study the viscosity-temperature relationship for the aqueous solutions of prepared polymers, attempting to establish the relationship between the molecular structure of the polymers and the thermoviscosifying properties. Hopefully, it can provide the theoretical basis of such polymers for the further industrial development and application.

#### 3.2.1. Effects of Concentration on Thermoviscosifying Behaviors

**Figure 6:**
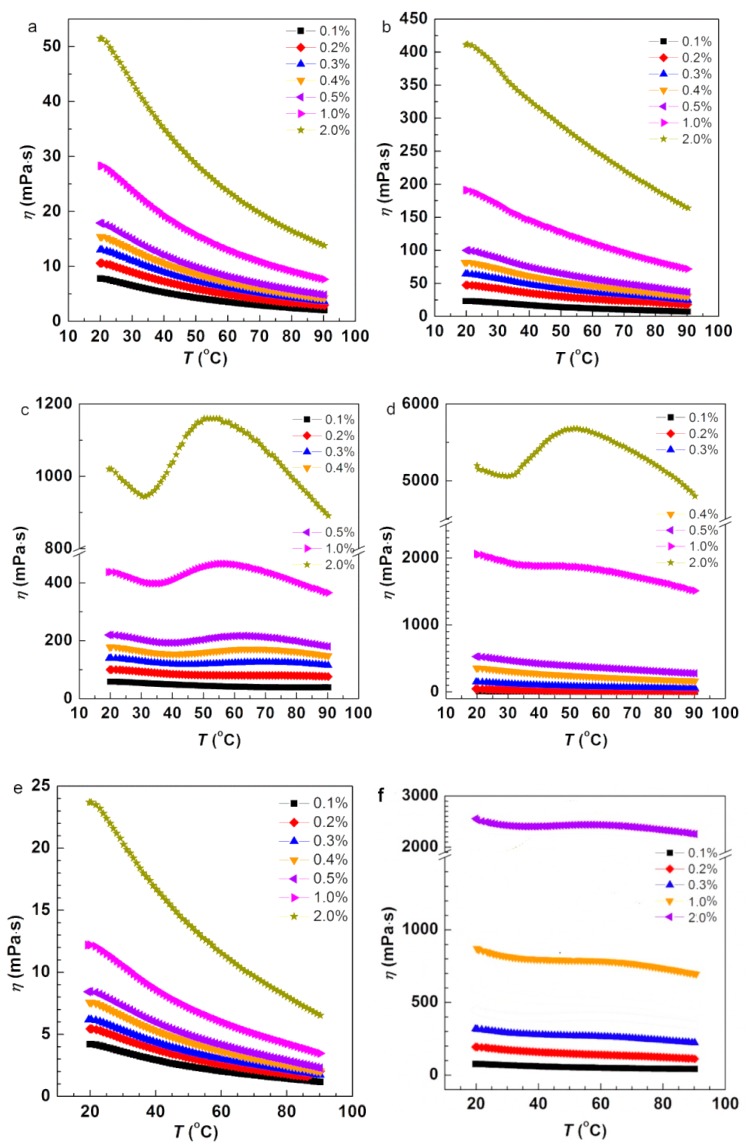
Apparent viscosity plotted as a function of polymer concentration for (**a**) S1, (**b**) S2, (**c**) S3, (**d**) S4, (**e**) S5 and (**f**) unmodified Pluronic F127 in pure water (shear rate = 10 s^−1^).

**Figure 7:**
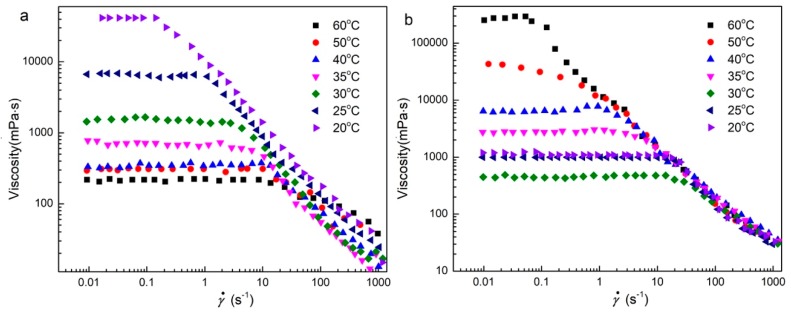
Rheological behavior of (**a**) S2 and (**b**) S3 with different shear rate and temperature. *C*_p_ = 2.0 wt %.

From Figure 6, it can be seen that under the same temperature, viscosity of the polymer solutions intensifies with the increasing concentration. After the concentration of the polymer solutions increases to a certain extent, some polymers have the thermoviscosifying behaviors, i.e., the viscosity of polymer solution increased with raised temperature rise. Thermal association temperature (T_ass_, the corresponding temperature when viscosity begins to rise) decreased with the increased concentration. For instance, the thermal association temperature was 45 °C when the concentration of S3 was 0.3 wt %, but T_ass_ of S3 was changed to at 1.0 wt % and it decreased to 31 °C at 2.0 wt %. The polymers S3 and S4 have T_ass_, and both of them are modified Pluronic F127 with NaAMPS and AM; *T*_ass_ decreased with the increase of concentration, and the reason is that higher polymer concentration is conducive to form a physical crosslinking network structure. Moreover, the viscosity values of different polymers at the same concentrations are quite different, and the reason may be that the viscosity is affected by the molecular weight of the main chain and the grafting ratio; however, more experiments should be done to prove this. Figure 7 shows the rheological behavior of S2 and S3 with different shear rate and temperatures. Figure 7a demonstrates that the enhanced temperature decreased the viscosity of S2 solution, but S3 has a thermoviscosifying behavior. The viscosity at platform maintains a good linear relationship with critical shear rates. The enhanced temperature significantly improves the shear thinning. When the shear rate reaches a certain level at different temperatures, the apparent viscosity of polymer solutions remains a certain value, indicating that increasing temperature struiopongly promotes the expansion of the association region.

#### 3.2.2. Effects of Molecular Structure on Thermoviscosifying Behaviors

Thermoviscosifying performances of S1, S2, S3, S4 and S5 were compared under five different concentrations. For quantitative comparison of thermal viscosity enhancements, two parameters were chosen. The first one is *η*_max_/*η*_30_, which is the ratio between the maximum viscosity and the viscosity at 30 °C, representing the increment of increased viscosity. The second parameter is T_ass_.

**Figure 8:**
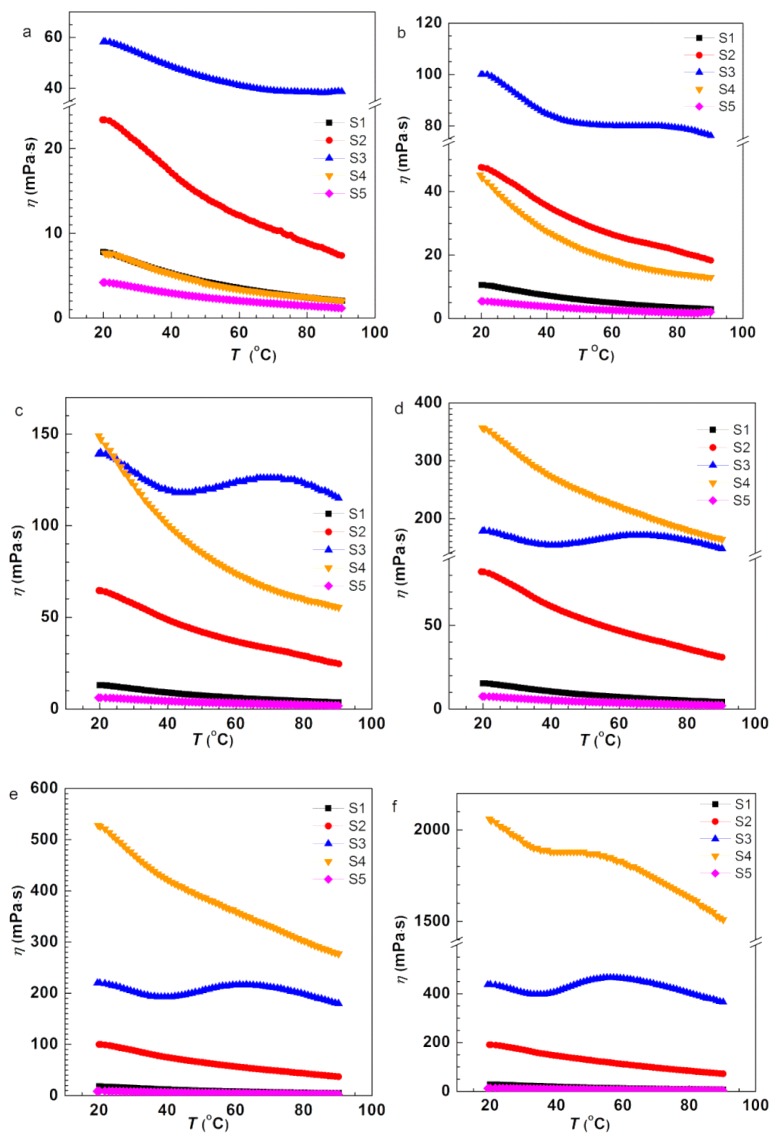

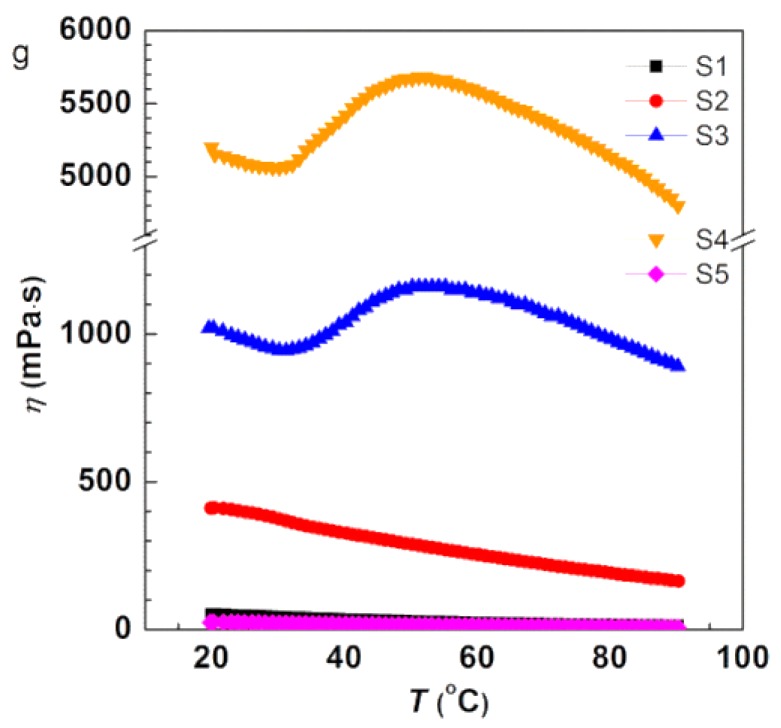
Apparent viscosity plotted as a function of temperature for S1, S2, S3, S4 and S5 at the same concentration in pure water: (**a**) 0.1 wt %; (**b**) 0.2 wt %; (**c**) 0.3 wt %; (**d**) 0.4 wt %; (**e**) 0.5 wt %; (**f**) 1.0 wt %; (**g**) 2.0 wt % (shear rate = 10 s^−1^).

**Figure 9:**
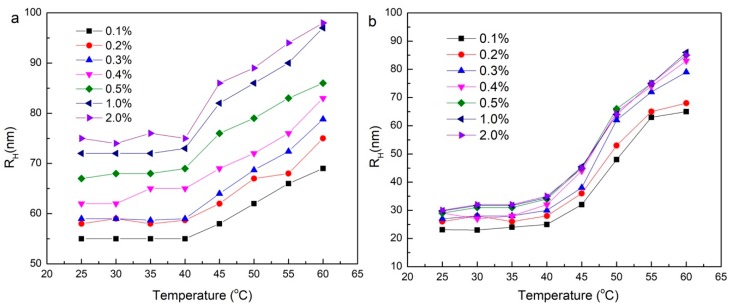

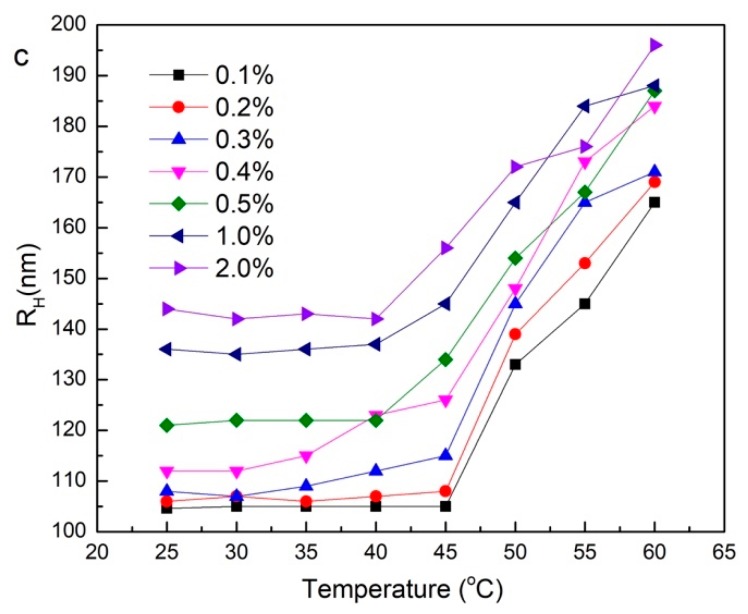
The hydrodynamic radius (R_H_) versus the temperature for S2, S3, S4 aqueous solutions with different concentrations and temperatures, respectively.

Figure 8 and Table 4 indicate that although the M_w_ of S4 exceeds 10 million, the viscosity of S4 solution is lower than that of S3 when C_p_ < 0.3 wt %. The reason is that NaAMPS content in S4 is much smaller than that in S3. The abundant anion groups from NaAMPS cause mutual repulsion between groups, thus extending the spatial structure of chain easily and it is manifested by high viscosity macroscopically. Moreover, S4 shows the highest viscosity when C_p_ is higher than 0.3 wt % and the viscosity of S4 solution reaches 5500 mPa⋅s at 2.0 wt %. It is displayed that viscosity of polymer solution is positively related with molecular weight. Additionally, thermal viscosity enhancement occurs in S3 when the concentration is 0.3 wt %, but thermal viscosity enhancement occurs in S4 only when the concentration is 2.0 wt %. This is attributed to the higher content of short hydrophobic chains PPO in S3 which develop the thermal viscosity enhancement. Therefore, S3 is easier to produce hydrophobic interaction and forms the hydrophobic association network. The thermal association temperature is lower. Furthermore, for S3 and S4, their thermoviscosifying behaviors occur at 2 wt % and their thermal association temperatures are same (31 °C). However, the increment of increased viscosity for S3 is larger than that of S4. Combining with their graft ratios and contents of Pluronic F127, it is speculated that these phenomena are caused by two reasons. Firstly, because B_S4_ > B_S3_, more side chains result in the stronger intermolecular interaction and the higher viscosity. For S2, S3 and S4, their fractions of PPO for S2, S3 and S4 are almost the same, and their molar ratios of AM/NaAMPS are quite different. It is believed that the molar ratio of AM/NaAMPS for S3 is about 3, which causes S3 to have good thermoviscosifying behavior. Secondly, PPO block in Pluronic F127 has its own LCST and the content of PPO block in S3 is higher than that in S4. With the increase of temperature, PPO block develops hydrophobic association, causing increased viscosity in the macroscopic view [26,27,28]. Figure 9 demonstrates the hydrodynamic radius obtained from DLS was enhanced with increasing temperature and concentrations [29,30,31]. DLS can show the distribution of sizes for polymer aggregates, and hydrodynamic radius was obtained from the peak value of size distribution. For example, Figure S2b and Figure 9a both show the hydrodynamic radius of S2 in water (0.1 wt %) at 25 °C was 55.3 nm.

## 4. Conclusions

Five polymers with different molecular structures are prepared through graft polymerization of Pluronic F127 with different concentrations of AM and NaAMPS. Molecular structures of these five polymers were tested through FT-IR and NMR. The molecular structure-thermoviscosifying relationship was established. The higher PPO content causes the stronger thermoviscosifying performance. Apparent viscosity of polymers is determined by M_w_, content of anion groups and graft ratio. Viscosity of polymers is positively related with M_w_, content of anion groups and number of side chains. S2, S3 and S4 have the same fractions of PPO, but their molar ratios of AM/NaAMPS are quite different. The molar ratio of AM/NaAMPS for S3 is about 3, which causes S3 to display good thermoviscosifying behavior. When the polymer concentration is higher than the critical value, viscosity of polymer solution increases with increasing M_w_. The micelle size of the prepared polymers in aqueous solutions was also investigated by DLS, and the micelle size increased with increasing temperatures and concentrations.

## Figures and Tables

**Table 1:** Feed weight, yield and chemical formula of the five prepared polymers.

Sample	Feed Weight			Yield (%)	B ^c^ (%)	Chemical Formula
	F127	NaAMPS ^a^	AM ^b^			
S1	20.02 g (1.5 mmol)	16.03 g (70 mmol)	/	83%	3.42	F127-NaAMPS_31_
S2	20.01 g (1.5 mmol)	12.01 g (52 mmol)	3.99 g (56 mmol)	81%	4.78	F127-NaAMPS_32_-AM_47_
S3	20.03 g (1.5 mmol)	8.02 g (35 mmol)	8.01 g (113 mmol)	89%	5.01	F127-NaAMPS_37_-AM_142_
S4	20.01 g (1.5 mmol)	4.02 g (18 mmol)	12.01 g (169 mmol)	84%	5.72	F127-NaAMPS_26_-AM_205_
S5	20.02 g (1.5 mmol)	/	16.02 g (226 mmol)	85%	6.61	F127-AM_300_

^a^ 2-Acrylamido-2-methylpropane sulfonic acid sodium salt; ^b^ acrylamide; ^c^ grafting ratio.

**Table 2:** Weight fraction of monomers in polymer and molar ratio of AM/NaAMPS for the five prepared polymers.

Sample	F127 (wt %)	NaAMPS (wt %)	AM (wt %)	AM/NaAMPS (mol/mol)
S1	63.9	36.1	0	/
S2	54.1	31.6	14.3	1.46
S3	40.4	27.3	32.3	3.83
S4	38.0	18.1	43.9	7.88
S5	37.2	0	62.8	/

**Table 3:** Structural parameters of the five polymers in mixed formamide/water (1:1) at 25°C.

Sample	A_2_ (×10^4^ mL·mol·g^−2^)	R_g_ (nm)	M_w_^a^ (×10^6^)	M_w,GPC_^b^ (×10^6^)	Đ ^c^	*K*_H_	[*η*] (mL⋅g^−1^)
S1	9.87	32.1	0.24	0.27	2.98	0.52	501.9
S2	4.34	66.9	2.08	2.18	2.87	0.56	591.3
S3	8.86	34.9	0.62	0.68	2.95	0.34	651.2
S4	0.642	131.5	15.50	/ ^d^	/ ^d^	0.34	715.3
S5	5.43	56.2	1.17	1.36	3.08	0.34	527.7

^a^ Molecular weight measured by SLS; ^b^ molecular weight measured by GPC; ^c^ dispersity of molecular weight obtained by GPC; ^d^ cannot be tested by GPC.

**Table 4:** Thermoviscosifying ability of different polymers at five different concentrations.

		*η*_max_/*η*_30_					*T*_ass_ (°C)				
	Sample	S1	S2	S3	S4	S5	S1	S2	S3	S4	S5
C_p_ (wt %)											
0.3		/^a^	/	1.01	/	/		/	45.0	/	/
0.4		/	/	1.04	/	/	/	/	42.0	/	/
0.5		/	/	1.07	/	/	/	/	41.0	/	/
1.0		/	/	1.15	/	/	/	/	36.0	/	/
2.0		/	/	1.23	1.12	/	/	/	31.0	31.0	/

^a^ It does not exist.

## Supplementary Materials

The following are available online at https://www.mdpi.com/2073-4360/11/10/1702/s1.
