# Supplementary material for: The Thermoviscosifying Behavior of Water-Soluble Polymer Based on Graft Polymerization of Pluronic F127 with Acrylamide and 2-Acrylamido-2-methylpropane Sulfonic Acid Sodium Salt
Source: Polymers (Basel). 2019 Oct 16;11(10):1702. doi: 10.3390/polym11101702 (PMC6835222; doi:10.3390/polym11101702)
Supplement: Supplementary file 1 [file polymers-11-01702-s001.pdf]

Supporting Information for

The Thermoviscosifying Behavior of Water-soluble Polymer Based on Graft Polymerization of Pluronic F127 with Acrylamide and 2-Acrylamido-2-methylpropane Sulfonic Acid Sodium Salt

Hongping Quan [1,†], Luyao Xie [2,†], Xin Su [2,*] and Yujun Feng [2,*]

[1] Oil & Gas Field Applied Chemistry Key Laboratory of Sichuan Province, School of Chemistry and Chemical Engineering, Southwest Petroleum University, Xindu 610500 China

[2] Polymer Research Institute, State Key Laboratory of Polymer Materials Engineering, Sichuan University, Chengdu 610065 China

* Correspondence:
Xin Su: xinsu@scu.edu.cn; Tel.: +86–28–85408037;
Yujun Feng: yjfeng@scu.edu.cn; Tel.: +86–28–85408037

† These authors contributed equally

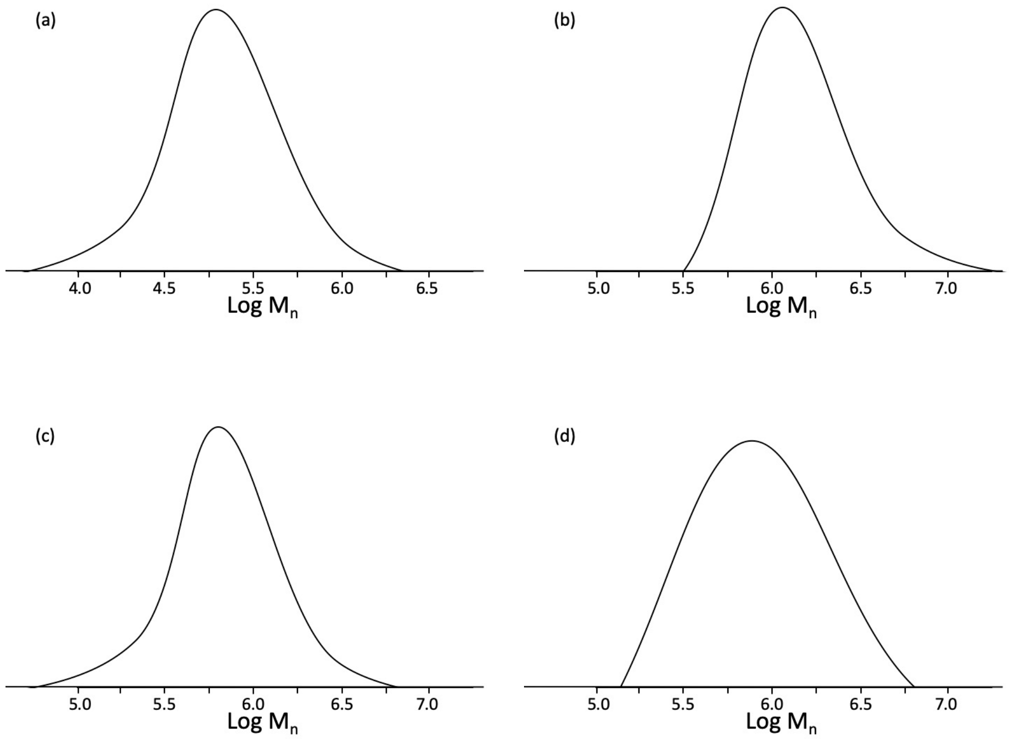

Fig. S1 Evolution of molecular weight distribution with conversion: (a) S1, (b) S2, (c) S3, (d) S5.

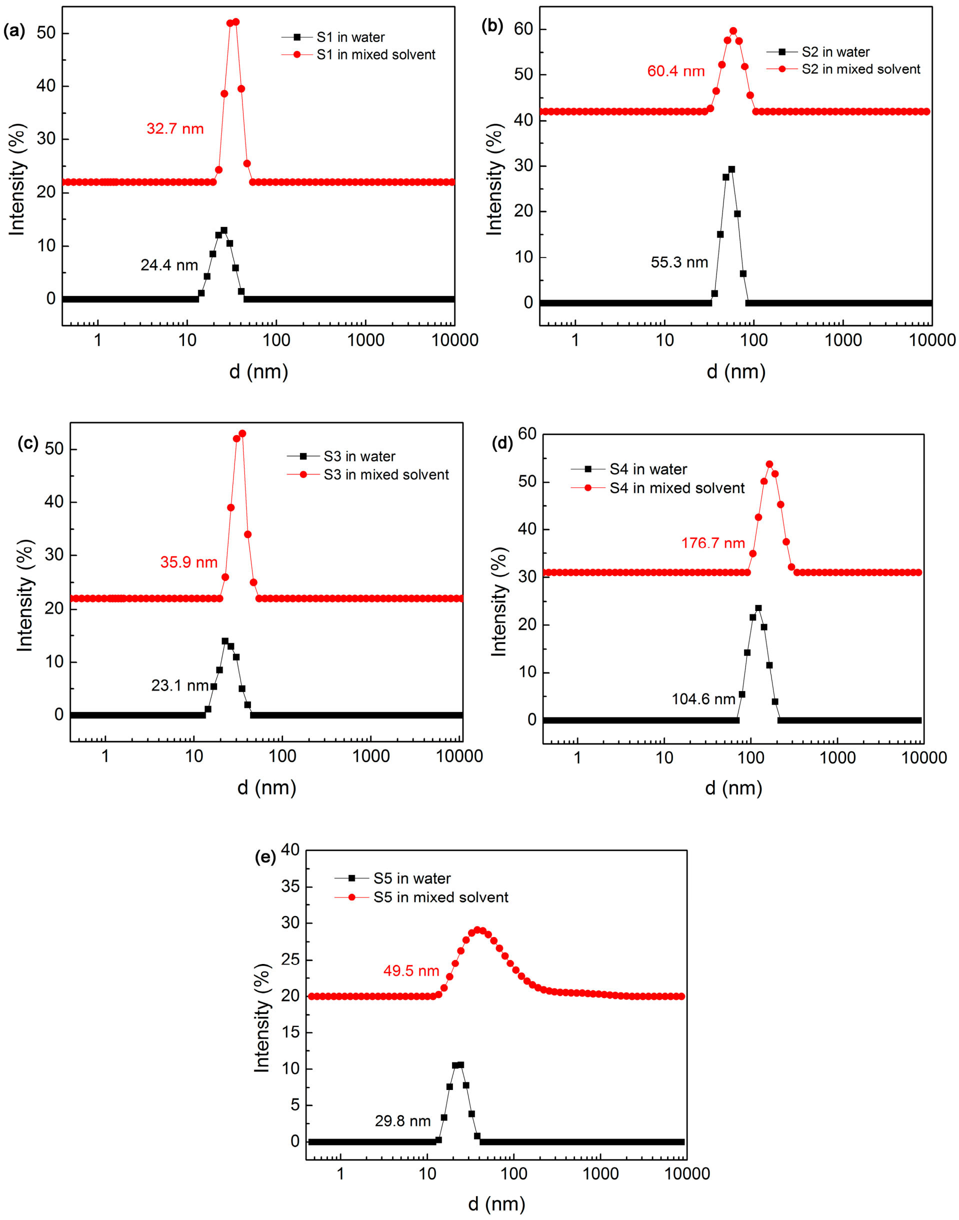

Fig. S2 DSL results of the polymers S1, S2, S3, S4 and S5 in pure water (red dots) and mixed formamide/water (1:1) at 25°C. The concentrations are 0.1 wt%.

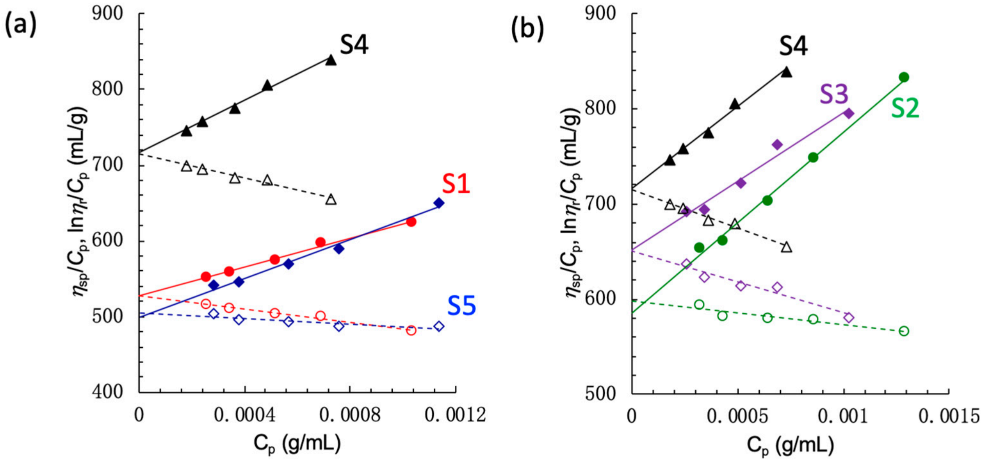

Fig. S3 η_{SP}/C_P and $\ln\eta_r/C_P$ plotted as a function of polymer concentration for (a) S1, S4, S5, and (b) S2, S3, S4 (Solvent: formamide; T=25°C).
